# Discussion on the possibility of multi-layer intelligent technologies to achieve the best recover of musculoskeletal injuries: Smart materials, variable structures, and intelligent therapeutic planning

**DOI:** 10.3389/fbioe.2022.1016598

**Published:** 2022-09-30

**Authors:** Na Guo, Jiawen Tian, Litao Wang, Kai Sun, Lixin Mi, Hao Ming, Zhao Zhe, Fuchun Sun

**Affiliations:** ^1^ Department of Computer Science and Technology, Tsinghua University, Beijing, China; ^2^ Institute of Precision Medicine, Tsinghua University, Beijing, China; ^3^ College of Engineering, China Agricultural University, Beijing, China; ^4^ Department of Biomedical Engineering, Tsinghua University, Beijing, China; ^5^ Musculoskeletal Department, Beijing Rehabilitation Hospital, Beijing, China; ^6^ Orthopaedics, Chinese PLA General Hospital, Beijing, China

**Keywords:** smart materials, variable structures, intelligent therapeutic planning, best recover of musculoskeletal, multi-layer intelligent technologies

## Abstract

Although intelligent technologies has facilitated the development of precise orthopaedic, simple internal fixation, ligament reconstruction or arthroplasty can only relieve pain of patients in short-term. To achieve the best recover of musculoskeletal injuries, three bottlenecks must be broken through, which includes scientific path planning, bioactive implants and personalized surgical channels building. As scientific surgical path can be planned and built by through AI technology, 4D printing technology can make more bioactive implants be manufactured, and variable structures can establish personalized channels precisely, it is possible to achieve satisfied and effective musculoskeletal injury recovery with the progress of multi-layer intelligent technologies (MLIT).

## 1 Introduction

**FIGURE 1:**
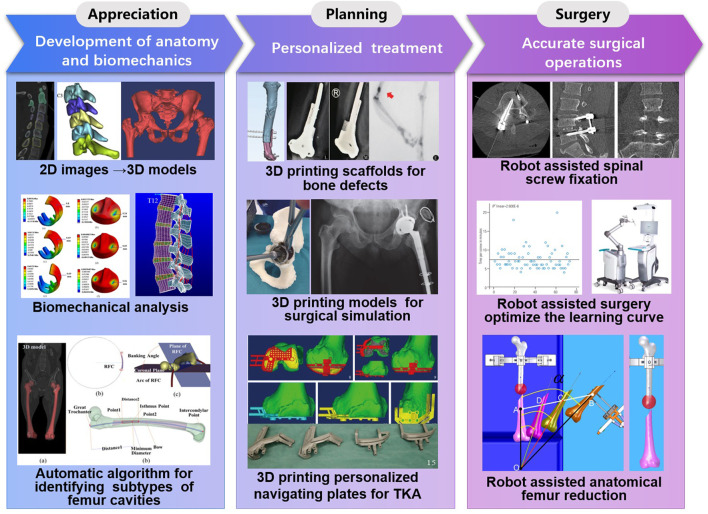
Intelligent technologies for precise orthopaedic.

In the past 30 years, intelligent technologies has facilitated the development of precise orthopaedic, an important direction in orthopedics/sports medicine, in the following three areas (Figure 1):

1) Digital orthopedics technologies have promoted the appreciation of digital anatomy (Pei and Yan, 2014) and biomechanics (Bhandarkar and Dhatrak, 2022). The three-dimensional models of bones reconstructed by digital orthopedics technologies, provide a means to establish the spatial relationship between anatomical structures. Shape statistical analysis allows that Elaborate anatomical analysis of bone can be performed. F. Chen Chen et al. (2018a) proposed a method to automatically identify three subtypes of femur cavities by k-means clustering analysis algorithm, on the basis of measuring the angle between the coronal plane and the radius of the femoral curvature (RFC) plane digitally. This method can provide a possible solution for the scientific design of Intramedullary (IM) nails, which will potentially facilitate IM nail implantation and reduce complications. Ghezlbash al. Ghezelbash et al. (2020) reviews the relevant findings of *in vitro* and finite element model studies on load-sharing in healthy, aged, degenerate and damaged human lumbar motion segments. They believed finite element model studies could improve understandings of functional biomechanics of human lumbar spine in normal and perturbed conditions.
2) 3D printing is an innovative technology for personalized treatment. Y. Liu et al. Liu et al. (2020) have prepared 3D printed polycaprolactone-hydroxyapatite (PCL-HA) porous scaffolds with loaded heparan sulfate (HS). This PCL-HA-HS scaffolds can accelerate the repairing of biological bone defects with sound compression resistance and good biocompatibility, which may be an effective biomaterial for bone defect repair. The study of B. Liu et al. Liu et al. (2021) showed that 3D printing technology can realize prosthesis stabilization and new bone regeneration in treating bone defects of limbs, to make patients achieve satisfactory limb function recovery. For total knee arthroplasty surgery, 3D printing navigation templates could predict prosthesis size accurately and provide an effective and precise guidance of osteotomy (Ding et al., 2017). 3D printing technology allowed accurate surgical simulation using life-size models, to valuate complex pelvic deformities precisely. This technology can improve anatomical appreciation and make personalized preoperative planning (Hughes et al., 2017), leading to reduce the risk of neurovascular injury.
3) Accurate surgical operations are carried out by robotics. In the past 5 years, the number of publications on orthopedic robotics has increased from 2500 to 6500 (Bernardo and Edoardo, 2021). Robot-assisted orthopedics surgeries can not only improve the accuracy of the operations (Jamwal et al., 2021), reduce operation time, radiation dose, and complications (Ye and Chen, 2009), but also optimize the learning curve (Nicolas et al., 2016; Kam et al., 2019; Tian et al., 2019).

However, simple internal fixation, ligament reconstruction or arthroplasty can only relieve pain of patients in short-term, because of the complexity of the musculoskeletal system (Neumann, 2010). The following problems limit the long-term therapeutic efficacy of precise orthopaedic:

1) Current surgical planning paths are the optimal geometric paths, but not the optimal bio-mechanical paths, which may not result in the optimal functional recovery of patients. The osseous tissue is not a uniform organization, which is mainly made up of two types of structural tissues, namely cancellous (trabecular) and cortical bone (Wubneh et al., 2018). The geometric center and the density center of the femoral head are different. Moreover, the density of different parts varies each other (Ahrend et al., 2021). Johns Hopkins University’s Vijayan R. Vijayan et al. (2019) reported an algorithm for automatic spinal pedicle screw planning using Active Shape Model (ASM) registration. But the manual path drawn by doctor is different with the automatic path calculated by the ASM algorithm. The fact that the clinical experiences, like predictions of bone mineral densities of different parts, may be considered into the manual preoperative planning by surgeons can result in the differences.
2) The fact that traditional bone grafts and bone substitute materials cannot provide signals for endogenous repair (Loebel and Burdick, 2018), can cause failure to induce bone formation or promote angiogenesis. Therefore, traditional orthopedics treatments may provide unsatisfied musculoskeletal rehabilitations. Bone grafts with shape memory effect, stimuli responsiveness, can maximize the new bones forming and the neovascularization, which are of great significance for improving the recovery of patients.

**FIGURE 2:**
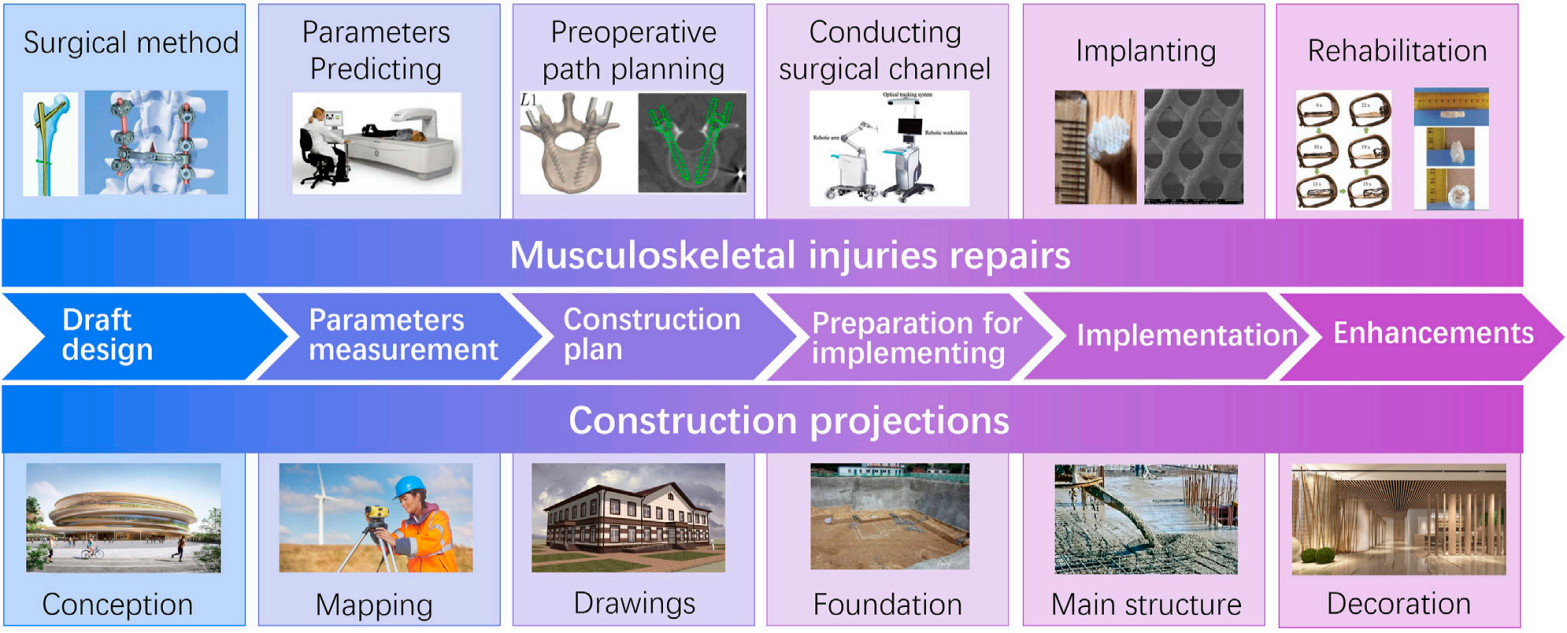
Comparison between construction projections and musculoskeletal injury repairs.

As shown in Figure 2, similarly to the construction process of a building, MLIT for musculoskeletal injuries consists of three stages: scheme design, scheme implementation, and enhancements (Table 1).

**TABLE 1:** Comparison between construction projections and musculoskeletal injury repairs.

**Stage**		**Construction projections**	**Musculoskeletal injuries repairs**
Scheme design	Draft design	Conception: Draw draft on basis of user requirements	Surgical plan: Select surgical method according to the patient’s condition, such as screw fixation, vertebroplasty, etc.
	Parameters measurement	Geographical mapping: Conduct field location-survey to get engineering mapping, including geotechnical, hydrology, etc.	Physical parameters prediction: Predict physical parameters of anatomical tissues, including bone mineral density, bone size, anatomical angle, etc.
	Construction plan	Construction drawings: Develop detailed construction plan according to the drafts and the mapping, including processes, materials, tools, etc.	Pre-operation path planning: Calculate surgical path on basis of predicted physical parameters and 3D reconstruction models, such as the position of the screw, the osteotomy plane, etc.
Scheme implementation	Preparation for implementing	Foundation building: Lay the foundation of the new building	Surgical channel establishment: Establish surgical channels with the use of a surgical robot
	Implementation	Construction of the main structure: Conducting the main structure on basis of construction plan, like reinforced concrete structures, etc.	Surgical procedure: Implant bone tissue engineering scaffolds
Enhancements		Decoration: Decorated the building according to the functional needs of users	Rehabilitation: Achieve satisfied recovery by staged stimulus action

Therefore, the multi-layer intelligent technologies (MLIT), including smart materials, variable structures, and intelligent therapeutic planning, which are on basis of the musculoskeletal biomechanical characteristics and the rehabilitation model of stimulus implants, will be the main trend of precise orthopaedic. Specifically, three bottlenecks must be broken through to achieve satisfied recovery: scientific path should be planned and built by AI algorithms, bioactive implants should be manufactured with smart materials, and personalized channels should be established by variable structures. This article will discuss the feasibility of satisfied and effective musculoskeletal injury recovery in the following aspects:

1) Scientific surgical path can be planned and built by through AI technology
2) Responsive bioactive implants made through 3D printing technology can provide signals for endogenous repair
3) Personalized surgical channels can be established through intelligent robotics precisely

## 2 Status of multi-layer intelligent technologies

### 2.1 Scientific surgical path can be planned and built by through AI technology

**FIGURE 3:**
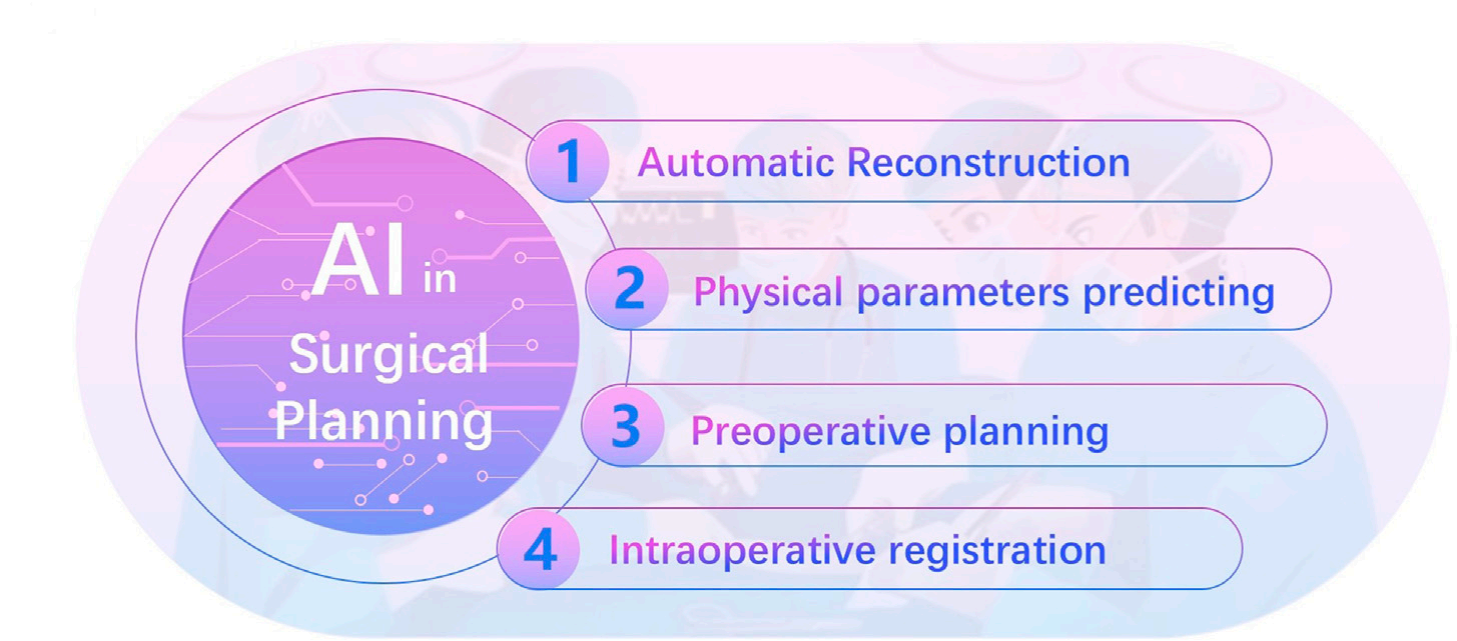
AI in surgical planning.

Surgical placement and appropriate implant fixation had equal importance as the inherent implant characteristics in maintaining long-term implant stability (Li et al., 2019). Biomechanical surgical path must be planned on basis of the pathogenesis of musculoskeletal diseases, to reach the satisfied musculoskeletal injury recovery. This requires physical parameters to be predicted precisely, to establish build kinesiology models of musculoskeletal system. In the past decade, AI technologies has made dramatic advances on Orthopaedic in the following four aspects (Zhou et al., 2020a; Lopez et al., 2021a) (Figure 3): automatic reconstruction, physical parameters predicting, preoperative planning and intraoperative images registration.

#### 2.1.1 Automatic reconstruction algorithm

**FIGURE 4:**
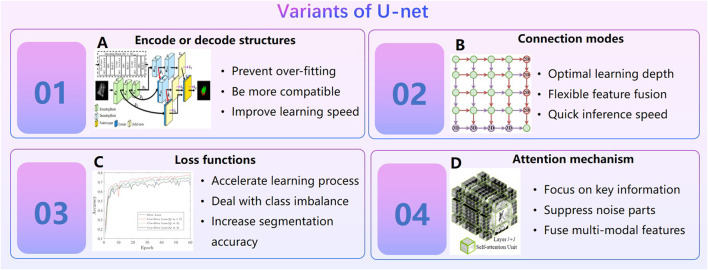
Variants of U-net. **(A)**. Cascade decoder: A Universal Decoding Method for Biomedical Image Segmentation (Liang et al., 2019). **(B)**. MNet: Rethinking 2D/3D Networks for Anisotropic Medical Image Segmentation (Dong et al., 2022). **(C)**. A Novel Focal Tversky loss function with improved Attention U-Net for lesion segmentation (Abraham and Khan, 2018). **(D)**. Swin UNETR: Swin Transformers for Semantic Segmentation of Brain Tumors in MRI Images.

In the early study, threshold segmentation or seed growth combined with manual repair are often used to segment medical images like CT, MRI etc. This semi-automatic method is very inefficient. AI technology, especially the U-net (Li et al., 2017; Falk et al., 2019), has promoted the rapid development of medical image segmentation. In general, variants of the U-net can be divided into four categories (Figure 4): ① Design encoder or decode structures. Cascade decoder conducted more effective decoding of hierarchically encoded features (Liang et al., 2019). The encoder of TernausNet removed the fully connected layers and replace them with a single convolutional layer of 512 channels that serves as a bottleneck central part of the network, and the decoder of TernausNet transposed convolutions layers that doubles thesize of a feature map (Iglovikov and Shvets, 2018). TernausNet was helpful to prevent over-fitting. ②Optimize connection modes of encoding module and decoding module. MNet proposed a skip connection method to balance the spatial representation inter axes *via* learning (Dong et al., 2022); UNet++ redesigned skip connections to exploit multiscale features in image segmentation (Zhou et al., 2020b).③ Set new loss function. Loss function is a method to measure the quality of model prediction, which is of course important to an AI model. Dice loss is a common loss function for medical image segmentation. However, it may cause oscillation during training when the prediction is close to ground truth (Chen et al., 2018b). Cos-Dice loss function was used in W-net to make the network more stable (Chen et al., 2018b). The adjustable penalty weights of the misclassified voxels were used in dice coefficient to adapt to unbalanced class frequency (Huang et al., 2018). Loss functions based on the Tversky index (Salehi et al., 2017; Abraham and Khan, 2018; Huang et al., 2018; Das and Zhang, 2020)were used to address the issue of data imbalance. ④Import attention mechanism. Swin Unet was a semantic segmentation of brain tumors in MRI Images using a swin transformer encoder which can extract features at five different resolutions by utilizing shifted windows for computing self-attention (Hatamizadeh et al., 2021). RA-UNet proposed a 3D hybrid residual attention-aware segmentation method to precisely extract the liver volume of interests (VOI) and segment tumors from the liver VOI (Jin et al., 2018). TransUNet was a variant of U-net using a transformer encodes which tokenized image patches from a convolution neural network (CNN) feature map as the input sequence for extracting global contexts (Chen et al., 2021). UNEt TRansformers (UNETR) utilized a transformer as the encoder to learn sequence representations of the input volume and effectively capture the global multi-scale information (Hatamizadeh et al., 2022). Particularly in 2021, the nn-Unet proposed by Isensee F. et al. Isensee et al. (2021) at Heidelberg University in Germany surpasses most existing approaches on 23 public datasets used in international biomedical segmentation competitions. Variants of the U-net or other deep learning algorithms are widely used in segmentations for pelvic, spine, femur, knee arthroscopy and other orthopedics fields (Zeng et al., 2017; Balagopal et al., 2018; Kolařík et al., 2019; Isensee et al., 2021).

#### 2.1.2 Physical parameters predicting method

Extraordinarily important is that AI technology perform well in predicting physical parameters of organizations. In 2019, a review by Rogers M.A. Rogers and Aikawa (2019) of Harvard University in the United States showed that AI technology can analyze the cardiovascular calcification on quantitatively large data sets. In 2020, Black K.M. et al. Black et al. (2020) of the University of Michigan automatically detected kidney stones composition from digital photographs of stones by ResNet-10, and precisions for each stone type were above 75%. In 2021, the study of Lopez F. et al. Lopez et al. (2021b) showed that the DCCN approach is the best method with a precision of 98% for four kidney stones classification with *in-vivo* endoscopic images. In 2021, Molnar D. Molnar et al. (2021) et al. of Gothenburg in Sweden proposed a Crop-Net for fat prediction, with a precision of 99.4%.

**FIGURE 5:**
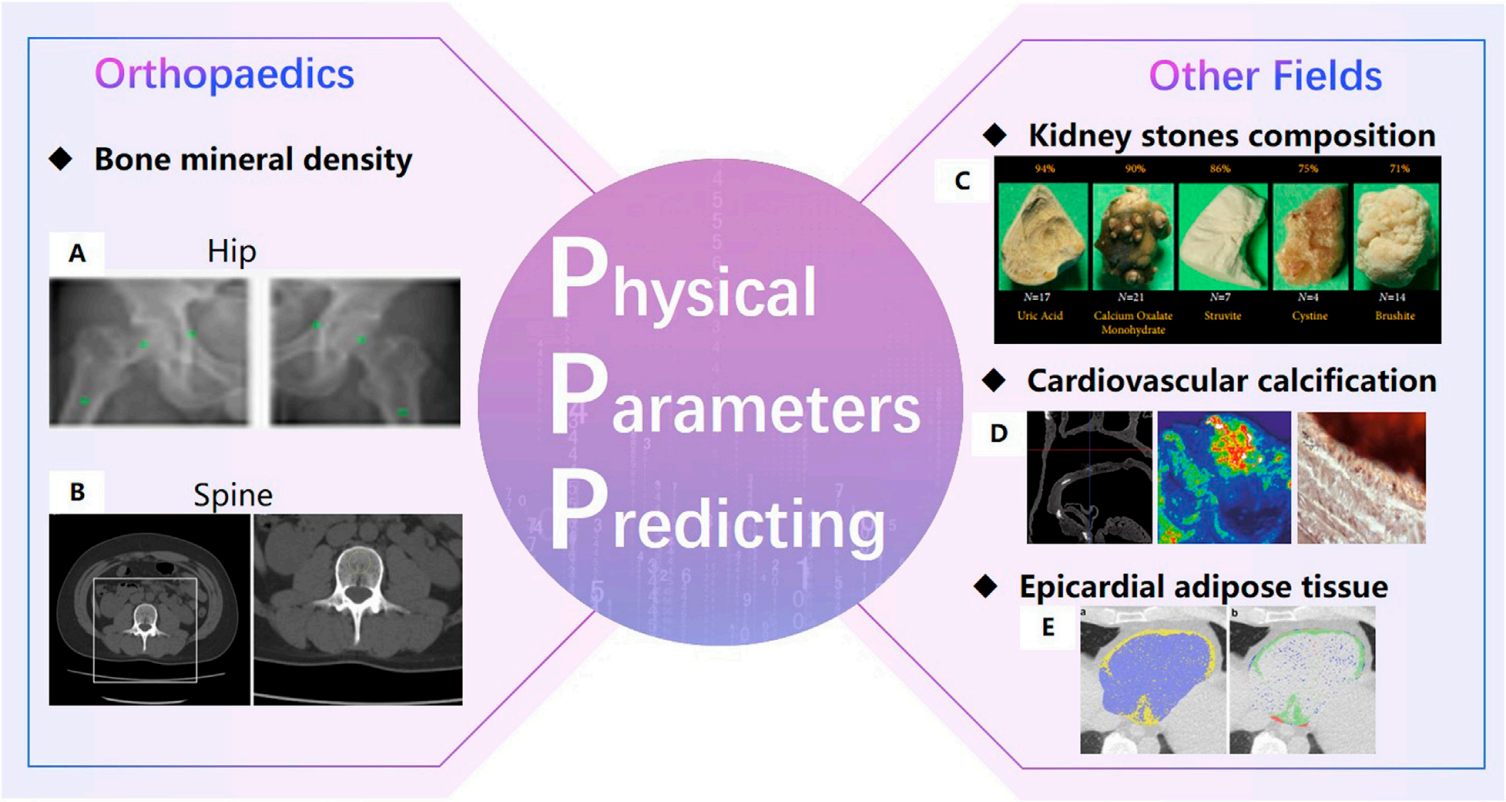
Physical Parameters Predicting (Hsieh et al., 2021). **(A)**. Bone mineral density prediction of hip (Yasaka et al., 2020). **(B)**. Bone mineral density prediction of lumbar spine. **(C)** automatically detect kidney stones composition (Black et al., 2020). **(D)**. cardiovascular calcification (Dhivya et al., 2015).**(E)**. Epicardial adipose tissue prediction (Molnar et al., 2021).

In the field of orthopedics, AI technology can predict bone density accurately (Figure 5). Hsieh C.I. from Chang Gung Hospital in Taiwan (Hsieh et al., 2021) proposed the Dual-energy X-ray A bsorptiometry (DXA)to predict bone mineral density and fracture risk, and the accuracy of hip osteoporosis prediction reached 95%. In 2020, the BMDCNN is applied to predict the bone mineral density (BMD) of the lumbar spine by Yasaka K. Yasaka et al. (2020) from the University of Tokyo, and the internal and external AUC of osteoporosis AUC 0.965 and 0.970.

#### 2.1.3 Preoperative planning

**FIGURE 6:**
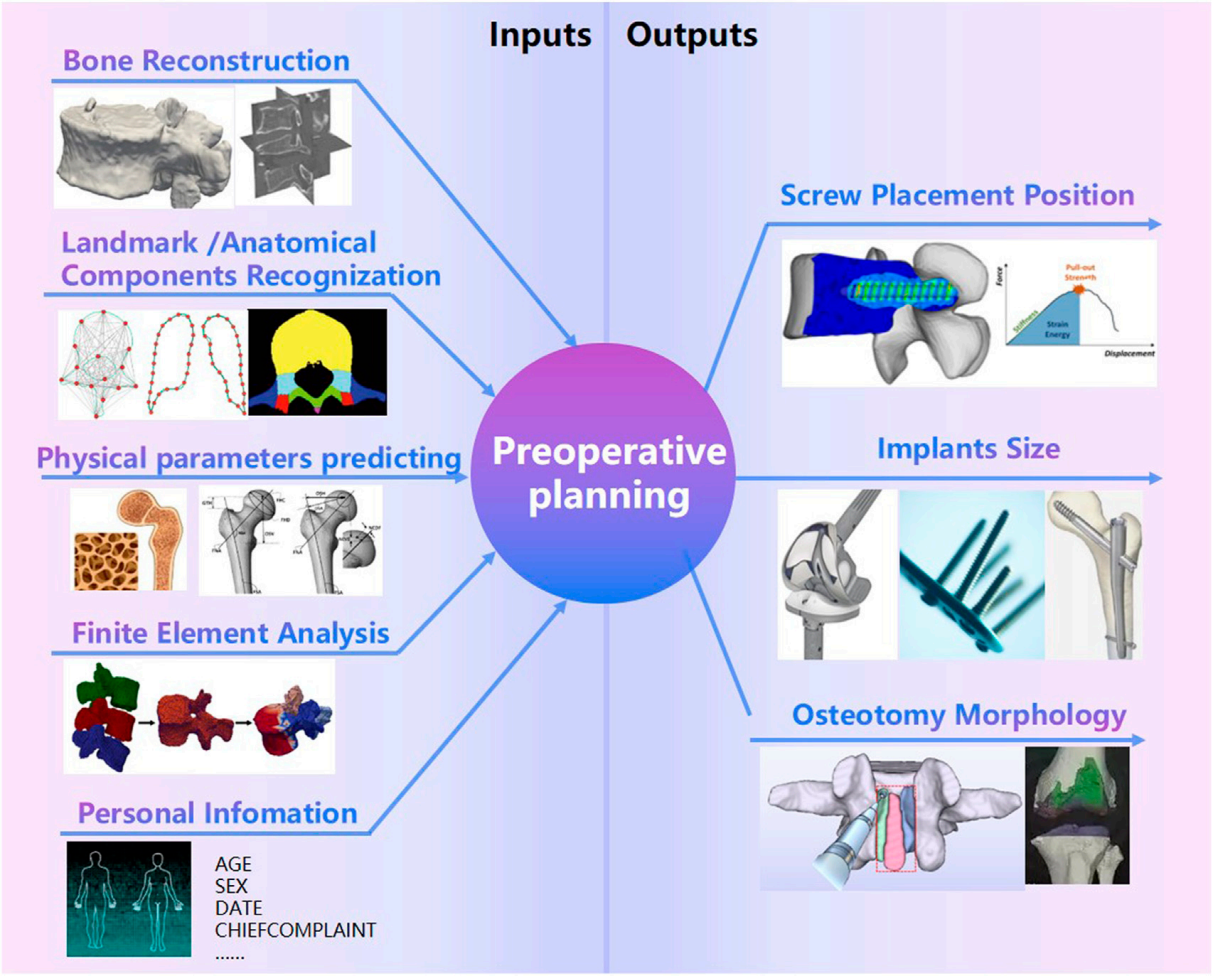
Preoperative planning.

Preoperative planning is an essential part of Clinical Decision Support System(CDSS). Screw placement position (Cai et al., 2019), implants size (Dong et al., 2021; Polce et al., 2021), osteotomy morphology are three common preoperative planning needs in Orthopaedic surgery (Figure 6). Target reconstructions, landmark/anatomical components recognization (Cai et al., 2019; Siemionow et al., 2021), physical parameters (bone mineral density (Caprara et al., 2021), morphological parameters (Soodmand et al., 2019)), finite element analysis (Zheng et al., 2018a; Caprara, 2021), personal information and other risk constraints may be possible inputs for preoperative planning. AI technologies can play a role in the processing of planning inputs. AI applications in bone reconstructions and physical parameters predictions have been discussed in Section 2.1.1 and 2.1.2. It is well-attended to predict how bone adapts to different loads in surgical planning (Jordi et al., 2022). FE is commonly performed in biomechanics analysis. It is necessary to build statistical shape models (SSM) based on a set of landmark points (Zheng and Yu, 2017) for FE analysis. An SSM built by the training dataset is a set of annotated images (Zheng and Yu, 2017). AI technologies make the landmarks/anatomical components recognization automatically (Siemionow et al., 2021). Nathan proposed a deep learning method based on the PointNet++ architecture for biomechanical modeling of facial tissue deformation in orthognathic surgical planning (Lampen et al., 2022). Xiao Xiao et al. (2021) proposed an estimating Reference Bony Shape Models for Orthognathic surgical planning using 3D point-cloud deep learning. Due to the incompleteness of information and uncertainty of healing prediction, preoperative planning dose not have a rapid development like reconstruction technologies.

#### 2.1.4 Intraoperative registration

**FIGURE 7:**
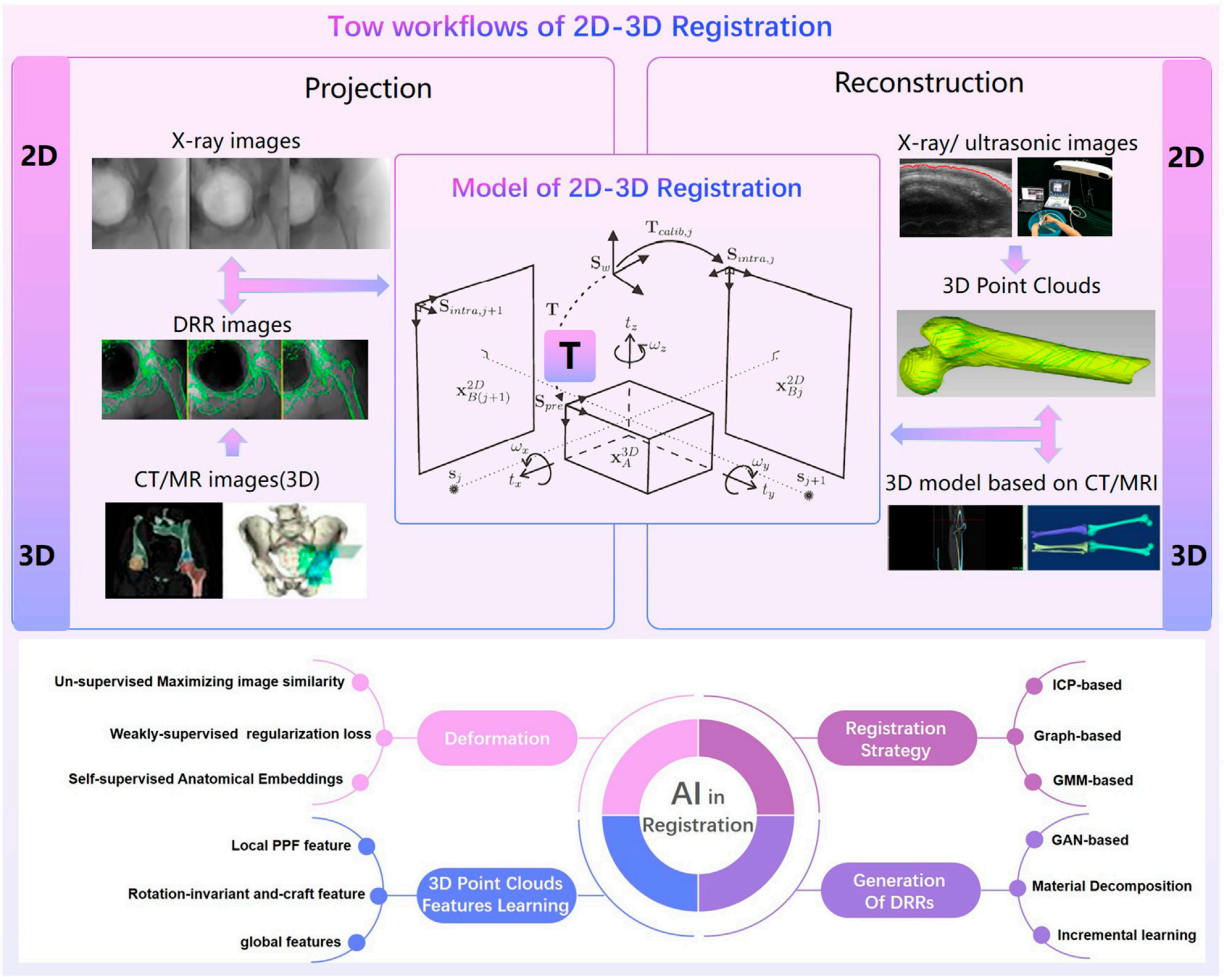
Tow workflows of 2D-3D Registration and AI in Registration.

The accuracy of robotic assisted surgery mainly depends on the space registration. The 2D-3D registration is commonly used in spinal surgery or pelvic surgery and the 3D-3D registration is commonly used in TKA (total knee arthroplasty) surgery. There are always tow workflows of 2D-3D registration (Figure 7): 1) Projection based 2D-3D registration. Digitally reconstructed radiographs (DRR) are generated from CT images, and then mutual information, normalized cross correlation, sum of square differences or other similarity measurements are used to calculate the relationship between X‐ray images and DRRs (Guo et al., 2019). 2) Reconstruction based 2D-3D registration. 3D point clouds are reconstructed from 2D x-ray or ultrasonic images. Transformation matrix are calculated through tow 3D point clouds (Wang et al., 2018). The intraoperative point clouds of knee are commonly collected by probes with markers in TKA surgery. The preoperative and intraoperative point clouds are matched through ICP or CPD algorithms.

Due to the sparsity inconsistency of point clouds between preoperative images and intraoperative images, deformation differences caused by different parameters of different imaging devices, the accuracy of surgical registration methods has been paid a great attention, but not yet been well solved. Registration methods based on deep-learning (Lu et al., 2021) have shown be capable of addressing the limitations of conventional registration methods (Figure 7), as they have a better performance in predicting deformations, learning 3D point clouds features, generating DRRs and optimizing registration strategy. Unsupervised learning (Chen et al., 2022) estimate voxel-to-voxel deformable transformation by maximizing image similarity (Fan et al., 2019). Weakly-supervised regularization loss is able to map thecomplex appearance to a common space (Blendowski et al., 2021). Self-supervised anatomical embedding (SAM) (Yan et al., 2022) is capable of computing dense anatomical/semantic correspondences between two images at the pixel level. Feature learning methods use the deep neural network to learn a robust feature correspondence search (Huang et al., 2021a), which includes a local PPF feature using the distribution of neighbour points (Deng et al., 2018), a rotation-invariant hand-craft feature (Gojcic et al., 2019), a global features (Qi et al., 2017) etc. There are three registration strategies using AI (Huang et al., 2021a): ICP-based variations, graph-based, GMM-based and semi-definite registration methods. Deep Closest Point(DCP) uses deep features to estimate correspondences to avoid spurious local optima of most ICP algorithms (Wang and Solomon, 2019). The surface registration are solved effectively by transforming the registration problem into a graph matching problem (Le-Huu and Paragios, 2017). For Gaussian mixture models (GMM), DeepGMR (Yuan et al., 2020) uses a neural network to learn pose-invariant point-to-distribution parameter correspondences. DRRs are simulated by algorithm which may differ from the real X-ray images. To improve the quality of DRRs, GAN-based training, material decomposition, and incremental learning are proposed. A GAN-based disentanglement learning framework (Han et al., 2021) can transfer the rib structural priors from DRRs. DeepDRR is a framework for DRRs embedding material decomposition and scatter estimation in 3D and 2D, combined with analytic forward projection and noise injection (Unberath et al., 2018). Incremental learning can improve continuously acquire new knowledge by continuously acquiring new knowledge (Jiang et al., 2021).

### 2.2 Responsive bioactive implants made through 3D printing technology can provide signals for endogenous repair

**FIGURE 8:**
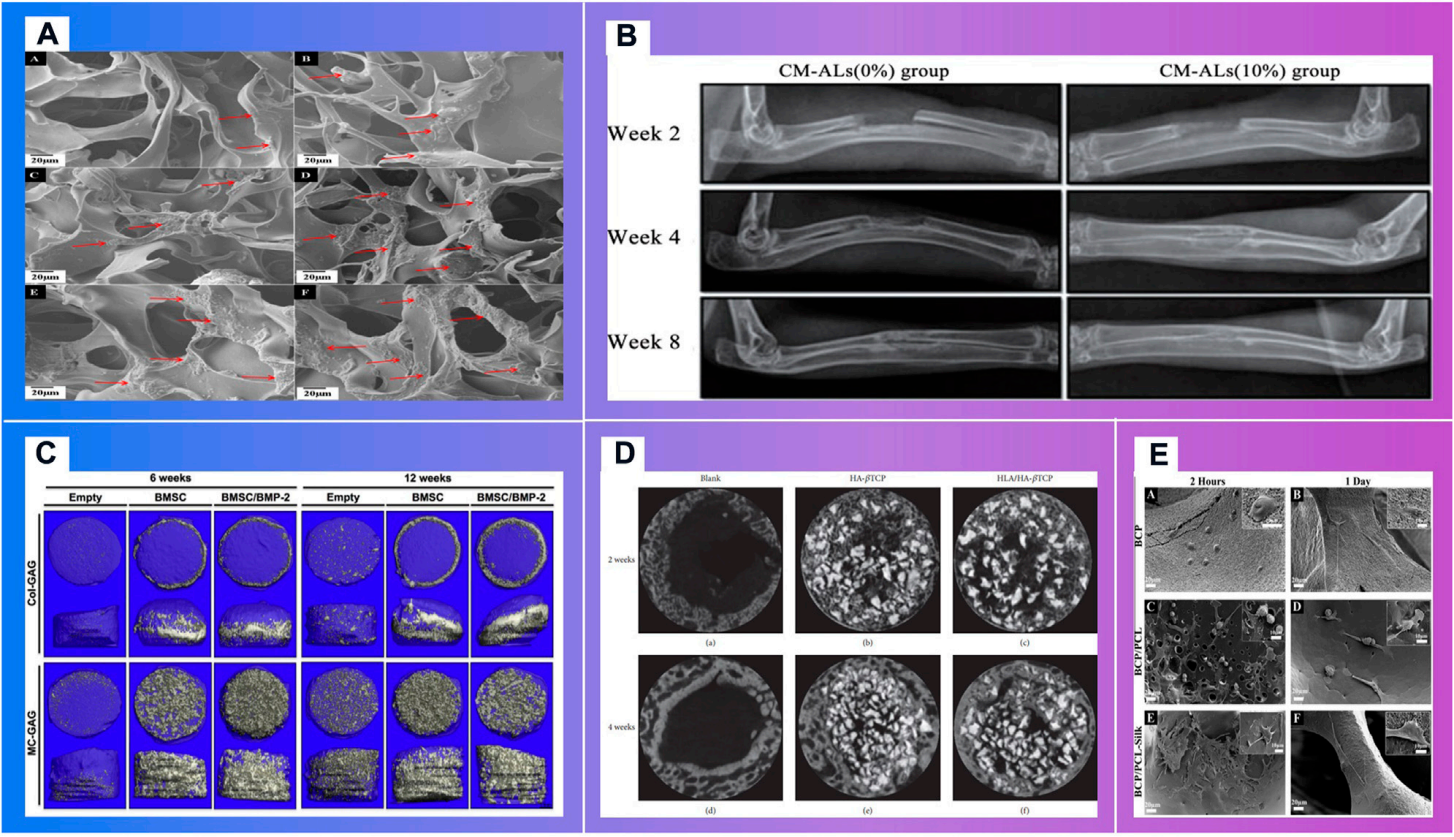
Bioactive materials demonstrating the osteoblast migrations. **(A)**. *In vitro* mineralization on mCT scanning of BMSCs cultured on Col-GAG and MC-GAG scaffolds in the absence and presence of BMP-2 (Makvandi et al., 2020) **(B)**. *In vitro* mineralization on mCT scanning of BMSCs cultured on Col-GAG and MC-GAG scaffolds in the absence and presence of BMP-2 (Ren et al., 2016) **(C)**. X-ray analysis of new bone formation in CM-ALs (0%) and CM-ALs (10%) implanted groups (Wu et al., 2017). **(D)**. Micro-CT images of the artificial defects at weeks 2 and 4. (Chang et al., 2016) **(E)**. Morphology of cultured HOB on **(A,B)** BCP, **(C,D)** BCP/PCL and **(E,F)** BCP/PCL–silk scaffolds after 2 and 24 h (Roohani-Esfahani et al., 2012)

Autograft, allografts and metal-based material scaffolds are commonly used in the clinical treatment of bone defects. However, chronic inflammation, immune rejection or stress shield and inflammation have hindered their clinical application. New bioactive responsive implants made through 3D printing technology and adaptively expanded, which can provide signals for endogenous repair, is highly necessary to promote bone regeneration (Figure 8). Bioactive materials, especially bioactive polymers high osteoinduction, excellent angiogenesis, biocompatibility and unlimited size, can interact with proteins, cells or tissues *in vivo* and cause biological reactions.

#### 2.2.1 Responsive bioactive materials can promote the reconstruction of organizations

**FIGURE 9:**
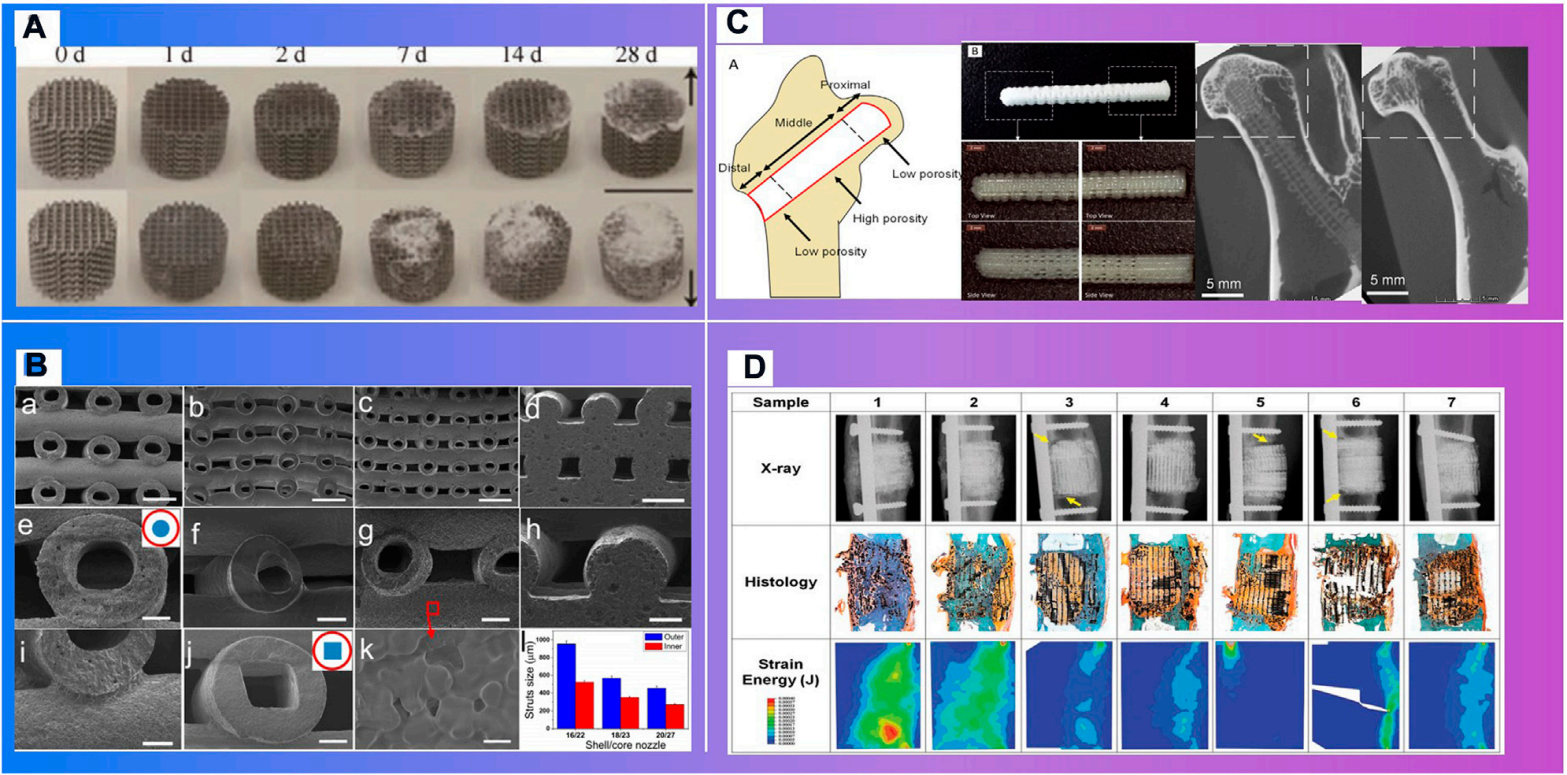
Materials with biodegradability. **(A)**. Degradation process (Li et al., 2020). **(B)**. SEM images of printed HSP bioceramic scaffolds *via* core/shell nozzle of 16/22 18/23 and 20/27 and printed SSP bioceramic scaffolds as the control (Luo et al., 2015). **(C)**. A functionally graded scaffold (Kawai et al., 2018). **(D)**. Mathematical modeling of total strain energy for defects implanted with the Sr-HT-Gahnite scaffold at 12 months postimplantation (Li et al., 2019).

Bioactive materials can guide bone regeneration (Mistry et al., 2015) (Figure 9). Dhivya S.et al. Dhivya et al. (2015) explored a nanoparticulate mineralized collagen glycosaminoglycan scaffold that induces healing of critical-sized rabbit cranial defects. These inorganic nano materials delivery of bioactive agents. However, their widespread employment may be reduced due to the possible toxicity and the lack of biodegradability (Makvandi et al., 2020).

Polymer materials consisting of inorganic nanomaterials and organic polymer materials, with high ductility, biocompatibility and biodegradability, are widely used in bone repair scaffolds (Figure 11). Their specific structure and surface properties can be specifically recognized and interact with target biomolecules. Makvandi P.et al. Makvandi et al. (2020) developed a graphene oxide (GO)–Chitosan (CS)–Hyaluronic acid (HA) based bioactive composite scaffold containing an osteogenesis-inducing drug simvastatin (SV). The elongated morphology of the cells after 48 h incubation has demonstrated the osteoblast migration using the SV loaded GO–CS–HA scaffolds. A collagen–hydroxyapatite(HA) scaffold (Unnithan et al., 2017) with a degree of interconnectivity of 99%, can not only deliver biological factors, promote stem cell differentiation and ossification (Thitiset et al., 2013; Villa et al., 2015), but also theoretically be applied in a load bearing application when combined with mechanical fixation (Zhang et al., 2018). Mouse BMSCs isolated from the femur and tibia of CD1 wild type animals, were seeded dropwise on to the top of either the Col–HA scaffold. Cells well were attached on the scaffolds after 12 h of seeding. In the study of *in vivo* bone formation in a mouse calvaria defect, scaffolds were implanted. After 3 weeks of implantation, calvaria containing critical size defects filled with BMSCs combined with either the Col–HA scaffold in X-ray images (Unnithan et al., 2017). The research of Ren X. et al. Ren et al. (2016) demonstrated that a nanoparticulate mineralized collagen glycosaminoglycan scaffold showed more efficient mineralization of MC-GAG scaffolds than non-mineralized Col-GAG scaffolds in either the histologic analyses or mCT scanning images. Wu H. et al. Wu et al. (2017) has fabricated a chitosan-based microsphere delivery system to controlled release of alendronate (AL), which can release AL for up to 30 days. CM-ALs (10%) scaffolds showed better performance in large-sized bone defects repairs than CM-ALs (0%). The new bone increasing ratio (NBIR) of HLA/HA-βTCP samples was 1.78 times higher than the blank group at week 2 (Chang et al., 2016). The study of Roohaniesfahani S.I. et al. Roohani-Esfahani et al. (2012) showed that, BCP/PCL scaffolds with silk layer is more favorable than BCP/PCL scaffolds with collagen layer in mechanical properties and biological properties.

**FIGURE 10:**
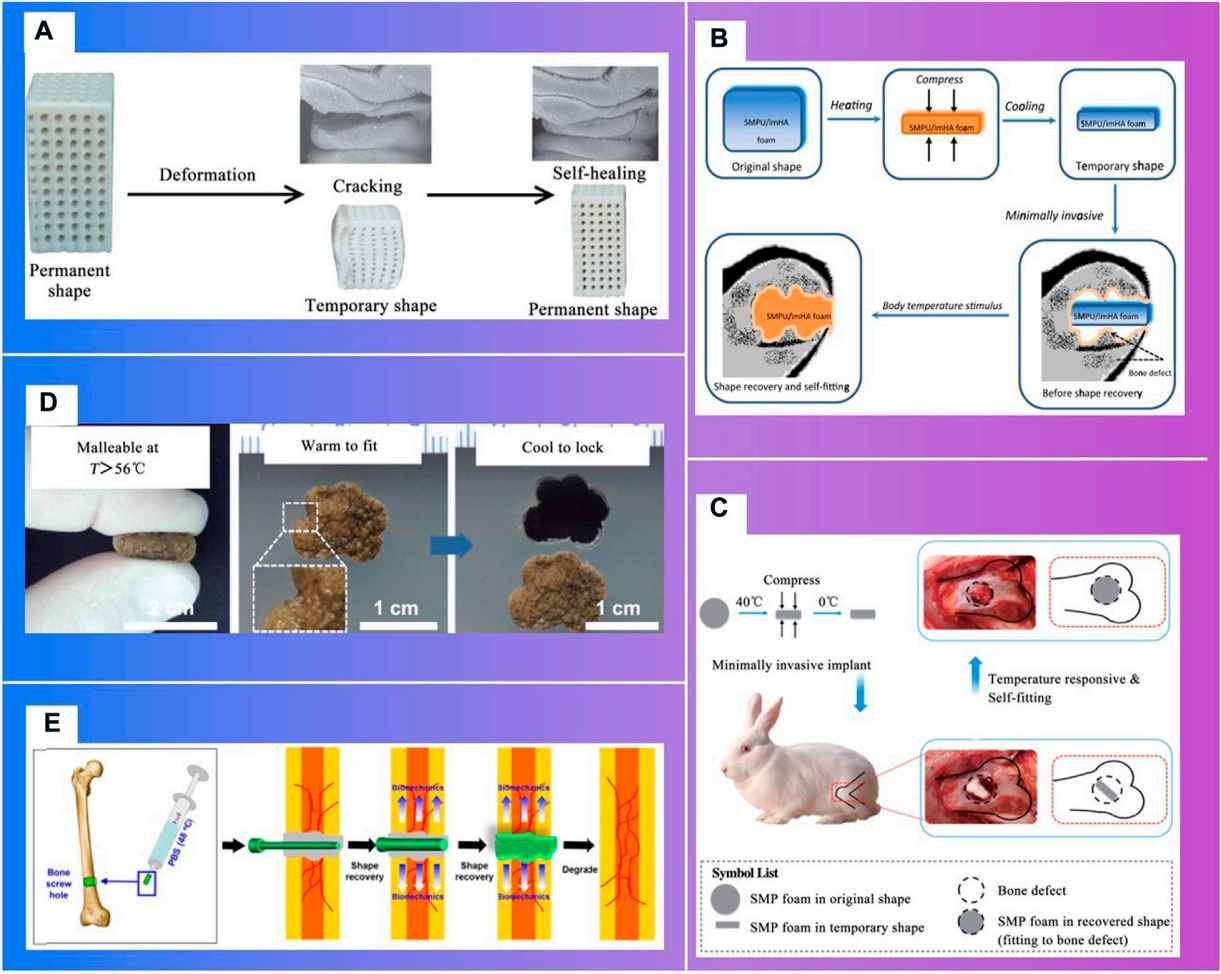
Shape memory polymers in bone tissue engineering. **(A)** t Fixing of a temporary shape of a 3D-printed PLA/HA scaffold through compression (Senatov et al., 2016) (Xie et al., 2017). **(B)**. Schematic of body-temperature responsive SMPU/imHA foam for minimally invasive delivery in application of bone regeneration (Xie et al., 2017). **(C)**. Schematic of self-adaptive SMP foam as a bone scaffold for bone regeneration (Xie et al., 2018). **(D)**. Self-adaptive process of polydopamine-coated shape memory porous PCL scaffolds (Zhang et al., 2014). **(E)**. Schematic illustration showing the application of shape-memory-capable scaffold of the Hap/PLMC naofibers (Xie et al., 2018).

More importantly, responsive bioactive materials, especially shape memory polymers (SMP) (Figure 10), can remember the temporary shape and return to the original shape under the condition of the external stimuli, like heat, pH, electricity, magnetic field, etc (Zhang et al., 2012; Rezwan et al., 2006; Zheng et al., 2018b). This specific shape memory could simplify complex transplant procedures, with excellent chemical stability, biocompatibility and biodegradability, which can stimulate specific cellular responses. Deng Z.et al. Deng et al. (2016) designed and synthesized a series of shape memory copolymers with electroactivity, super stretchability and tunable recovery temperature based on poly(e-caprolactone) (PCL) with different molecular weight and conductive amino capped aniline trimer. They proved that they can enhance myogenic differentiation from C2C12 myoblast cells. Xie R.et al. Xie et al. (2017) prepared a novel polyurethane or hydroxyapatite based SMP porous foam for the treatment of load-bearing bone defects by gas foaming. The foam can match the trabecular bone, possess the feasibility of minimally invasive delivery. And it can also overcome the disadvantages of traditional polymer foams in terms of insufficient mechanical properties, inadequate pore structures, low biocompatibility and inconvenience in operation. The rabbit femoral defect model (Xie et al., 2018) demonstrated that a SMP foam bone scaffold could play an important role in promoting neovascularization and bone remodeling. A shape memory PCL porous scaffold was made from photocrosslinking (ε-caprolactone) (PCL) polydiacrylate through SCPL method (Zhang et al., 2014), and it evidently promoted the adhesion and proliferation of osteoblasts. SMP scaffolds show strong self adaptability, as evidenced by the following facts. One of these is that smaller SMP scaffolds than bone defect size can match bone defect boundary after shape expansion (Zhang et al., 2012; Xie et al., 2017), the other is that larger SMP scaffolds can promote the inward growth of bone, due to the binding force with bone tissue (Zhang et al., 2014; Bao et al., 2016).

#### 2.2.2 3D bioprinting technology can personalize implants

**FIGURE 11:**
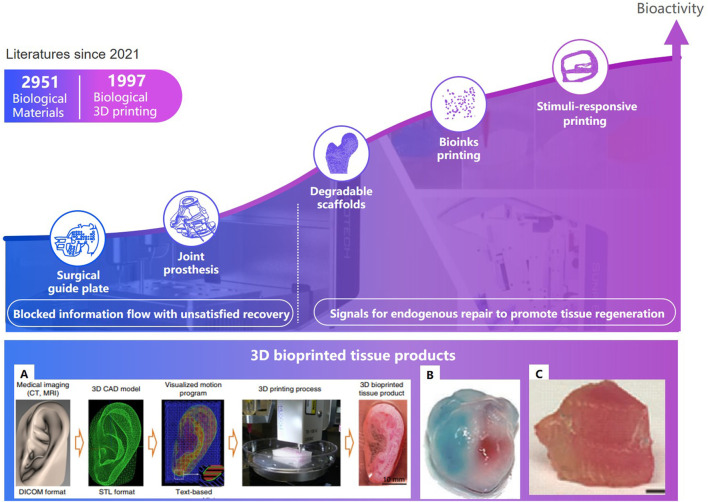
Development of tissue engineering and 3D bioprinted tissue product. **(A)**. The ITOP system (Groll et al., 2018).**(B)**. Ventricles (Zhang et al., 2016). **(C)**. Thyroid cartilage (Van Belleghem et al., 2020).

Three-dimensional printing technologies that can fabricate the microstructure of materials precisely, have shown distinct advantages to personalize implants in bone tissue engineering (Luo et al., 2015). In clinical medicine, 3D printing technologies can be divided into two stages (Figure 11): one is conventional 3D printing objects without cellular information exchange, like surgical guiding paltes (Butscher et al., 2013), prosthesis (Xu et al., 2013; Turnbull et al., 2018); the other is bioactive 3D printing objects can provide signals for endogenous repair, which particularly is of great significance to bone regeneration and vascular reconstruction, such as degradable scaffolds (Xu et al., 2019a), living cell printing (Kolesky et al., 2014), and stimuli-responsive printing materials with programmable behavior (Gladman et al., 2016) (Qi et al., 2014).

Degradable 3D printing scaffolds can gradually release the occupied space with the recovery of the damaged part (Figure 7.C) (Li et al., 2020). Kawai T. et al. Kawai et al. (2018) of Stanford University designed and 3D printed a functionally graded scaffold (FGS) made of polycaprolactone (PCL) and b-tricalcium phosphate (b-TCP) to treat Osteonecrosis of the femoral head. The *in vivo* degradation rate and the bone ingrowth ratio of the scaffold is significantly higher than the empty-tunnel group. Li et al. Li et al. (2019) at University of Sydney developed a 3D-printed Sr-HT-Gahnite scaffolds implanted into critical-sized segmental defects in sheep tibia. Compared with bone autografts, the scaffolds possessing both osteoconductive and osteoinductive properties, can induce substantial bone formation and defect bridging. Spiral fractures were observed in the study. The fractures may have a negative effect on the recovery. Thus, implants should be fixed at appropriate position.

#### 2.2.3 Bio implants through bio-inks can migrate in planned direction

Bioinks is a formulation of cells suitable for processing by an automated biofabrication technology that may also contain biologically active components and biomaterials (Groll et al., 2018). Biomaterials may consist of cells, collagen, and other bioactive ingredients; Auxiliary biomaterials, such as gelatin, alginate hydrogel, carbomer glue, etc, can be used to improve mechanical strength of implants, maintain the shape of the printed object, and ensure the adhesion and survival rate of cells. In 2015, Kang H.W. et al. Kang et al. (2016) of the Wake Forest Institute for Regenerative Medicine developed a system that deposits cell-laden hydrogels together with synthetic biodegradable polymers that impart mechanical strength. The printed mandible, skull, cartilage and skeletal muscle can incorporate multiple cell types at precise locations to recapitulate native structure and function. In 2016, Zhang Y. S. Zhang et al. (2016) of Harvard Medical School proposed a novel hybrid strategy based on 3D bioprinting to fabricate endothelializedmyocardium. Endothelial cells controlled anisotropy, can gradually migrate towards the peripheries of the microfibers.

With the continuous upgrading of material technology, biological 3D printing strategy is constantly updated. In 2019, Van Belleghem S.et al. Van Belleghem et al. (2020), from the University of Maryland College Park, proposed a 3D printing strategy for dual bioinks. The graft consisting of both degradable and nondegradable parts, providing long term mechanical integrity and shape retention. A pre-programmed responsive bioactive materials can be used in various application where the human intervention is not possible. This technology may lead to satisfied repair. The materials which responds to external stimuli is called 4D printing (Tibbits, 2014). A porous PLA/HAP scaffolds can stand up to three compression-heating-compression cycles without delamination. The scaffolds can narrow the cracks during heating, which may resulted in ‘self-healing’. The significant changes of post implantation have higher requirements to ensure the stability during deformation process of the implants, and that the deformation not cause additional damage to the surrounding tissues.

### 2.3 Personalized surgical channels can be established through intelligent robotics precisely

**FIGURE 12:**
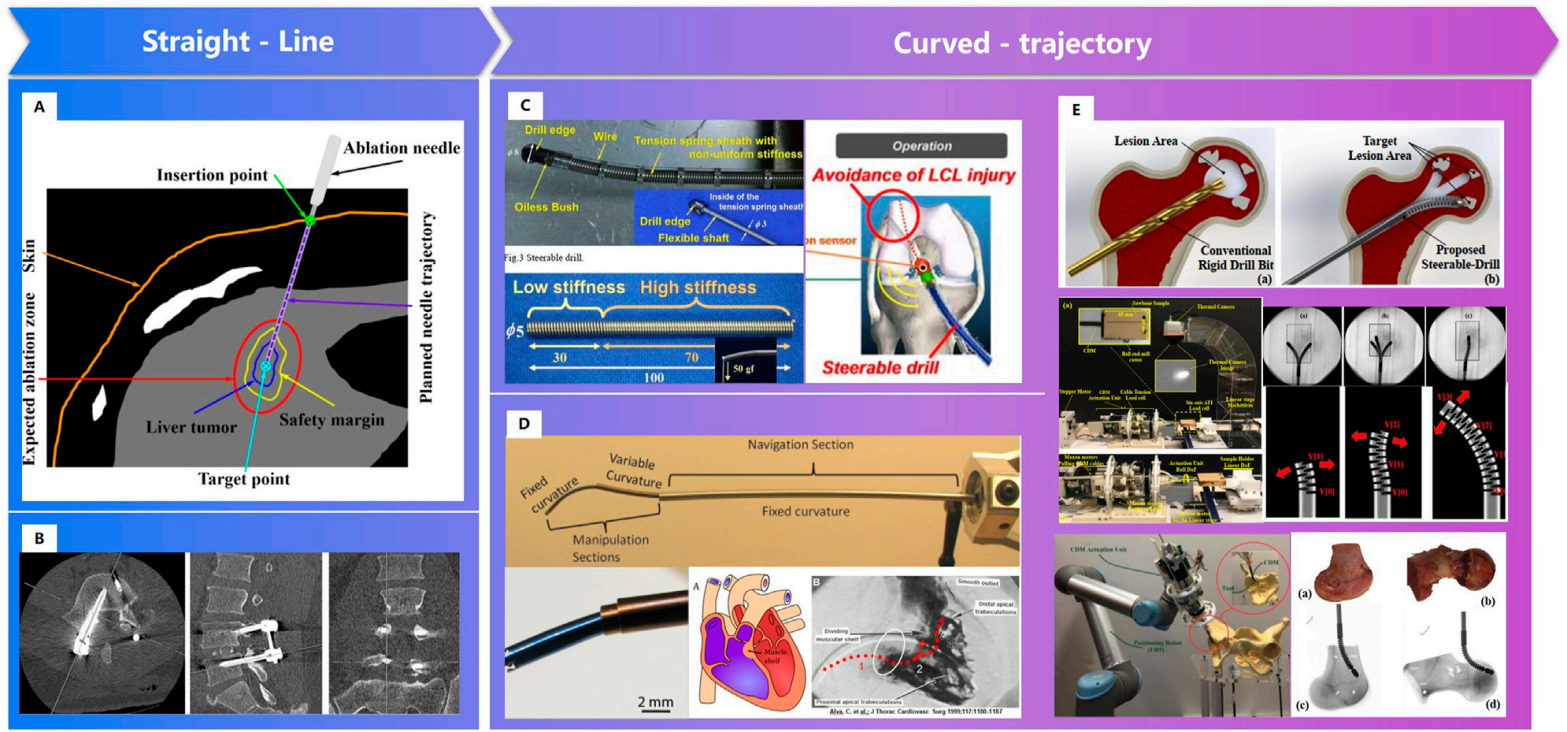
Surgical channels constructed by robot. **(A)**. Straight needle trajectory planning for radiofrequency ablation and microwave ablation of liver tumors (Zhang et al., 2019). **(B)**. Linear surgical channel for spinal screw internal fixation (Nicolas et al., 2016). **(C)**. A “Steerable Drill” for ACL Reconstruction to Create the Arbitrary Trajectory of a Bone Tunnel (DePhillipo et al., 2018). **(D)**. Endoscopic Add-on Stiffness Probe (Gilbert et al., 2014). **(E)**. A less-invasive surgical workstation to treat osteolytic lesions behind a well-fixed acetabular implant (Bakhtiarinejad et al., 2020)- (Alambeigi et al., 2014).

In clinical, there are linear channels established, due to the limitation of the structure of instruments (Nicolas et al., 2016; Zhang et al., 2019). Fracture or scoliosis treatments are great different between children and the elderly, because children have better self-healing in growth. Linear channels may be not meet the biomechanics needs, especially for the elderly. As smart materials and variable structures developed, such as memory alloys, concentric tube structures, etc., curved-trajectory, with less trauma and better therapeutic effect, attracts the researchers’ great attention (Figure 12). Moreover, intelligent sensing technology and intelligent operation technology also make the surgery more efficiency.

#### 2.3.1 Personalized surgical channels can be established through variable structures

Personalized surgical channels are linear channels, curved- channels or linear- curved mixed channels based on the status of patients. In 2011, Watanabe H. Watanabe et al. (2011) et al. developed a steerable drill for ACL reconstruction to construct an arbitrary trajectory of a bone tunnel. Gilbert H. Gilbert et al. (2014) adopted a needle-sized tentacle-like robot that require access through constrained paths in transnasal skull base surgery. In 2012, Gosline, A. H. et al. Gosline et al. (2012) manufactured a steerable curved concentric tube robot that can enter the heart through the vasculature, using a unique metal MEMS process.

Since 2014, Alambeigi F. of Johns Hopkins University et al. Alambeigi et al. (2014), Alambeigi et al. (2016a), Alambeigi et al. (2016b); Wilkening et al., 2017; Bakhtiarinejad et al., 2020) has researched the novel steerable drill using a continuum dexterous manipulator (CDM), and carried out experiments on both simulated and human cadaveric bones. Bakhtiarinejad M. Bakhtiarinejad et al. (2020) studied the use of curved drilling technique for treatment of osteonecrosis of femoral head. The biomechanical study demonstrated that a novel robot-assisted curved core decompression (CCD) technique is introduced to provide surgeons with direct access to the lesions causing minimal damage to the healthy bone. The progress of bistable structure, shape memory polymer and intelligent variable structure promoted the adaptability of surgical robot, which enable the biomechanical channels for orthopaedics.

#### 2.3.2 Intelligent sensing technology can lead to precise trajectory in complex environment

**FIGURE 13:**
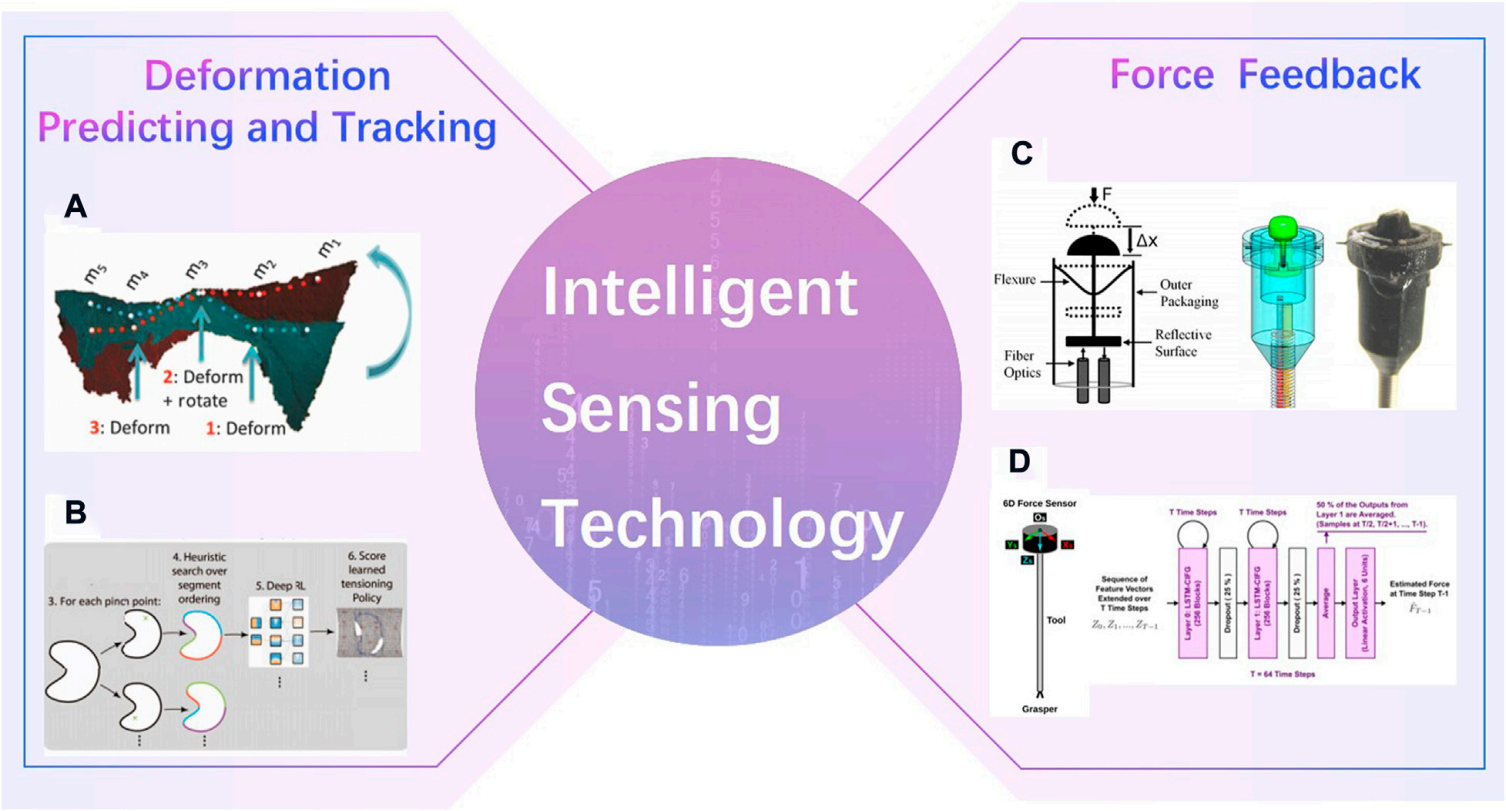
Intelligent Sensing Technology. **(A)** *in vivo* supervised autonomous soft tissue surgery (Shademan et al., 2016) **(B)**. Deep reinforcement learning policies were used in Pattern Cutting task (Thananjeyan et al., 2017). **(C)**. Force sensors in robotic surgery (Kesner and Howe, 2011) **(D)**. Semi-Supervised Deep Neural Network Model (Marban et al., 2018).

Unlike industrial scene, the intraoperative environments are always complex. The robot cannot perceive, explain and understand the surrounding environment as well as surgeons is one of the key factors that the robotics ca not perform surgery automatically. Deformation predicting and tracking, and haptic feedback are tow common intelligent sensing technologies in surgical robotics (Figure 13). Shademan Shademan et al. (2016) demonstrated *in vivo* supervised autonomous soft tissue surgery which can suture a wound automatically. Deep reinforcement learning policies were used in Pattern Cutting task to induce tension in the material as cutting proceeds (Thananjeyan et al., 2017). Force sensors were developed to estimate interaction forces in robotic surgery (Kesner and Howe, 2011; Marban et al., 2018). And a Semi-Supervised Deep Neural Network Model was applied to understand the operation pattern (Marban et al., 2018).

#### 2.3.3 Robotic skill learning can improve the automation level of surgical robotics

The surgical robotics in clinical are commonly semi-automatic. They can perform intraoperative actions based on the double closed-loop control of force position mixing in puncture (Wells et al., 2016), cochlear implant (Wang et al., 2020), fracture reduction (Lei et al., 2019) or other surgeries. However, it is impossible to dynamically adjust the task strategy according to the actual situation (Graham et al., 2008; Zhang, 2018). Skill learning (Silver et al., 2016; Mahler et al., 2019) make robots execute task which difficult by traditional control methods (Levine et al., 2016; Sun et al., 2017; Mandlekar et al., 2018), like pouring water and screwing screws, automatically.

## 3 Future research directions for satisfied repair of musculoskeletal injuries

The multi-layer intelligent technologies (MLIT) offer possibilities to achieve satisfied repair of musculoskeletal injuries. However, there are still no wide range of clinical applications, because of the following reasons: 1) the pathogenesis like the femoral head necrosis or the spinal tumor, the healing process like the ligament or the bone repair, and the biomechanical or kinematics models are not clarified in detail. 2) the real environment is hard to simulate. As there are differences between the simulation environment and the real environment, further verification of some research results should be implemented. 3) some theoretical designed surgical plan cannot be realized as expected. Further research will be needed for satisfied repair of musculoskeletal injuries:

### 3.1 The research on the pathogenesis or the healing process can be more effective through new AI technologies

#### 3.1.1 Biomechanical models based on multi-physical parameter prediction will be built

Current biomechanical models are always based on 3D reconstructed models. However, morphology and parameters of the ligament, meniscus (Antico et al., 2020) and other tissue are always artificially devised (Viris et al., 2016), and the same density of different bone tissues like cortical bone, cancellous bone and trabecular bone structures are commonly set in biomechanical analysis. It is said that kinematics simulations are greatly different from real scene (Bell et al., 1996). To build real biomechanical models, physical parameter such as the bone density, ligament elastic modulus, must be predicted precisely.

Although some physical parameters predicting methods are proposed, which are discussed in Section2.1.2, lots of parameters still cannot be predicted for the lack of data with high-quality labels. Standard labeling procedure, and cross check of data labels are necessary.

#### 3.1.2 Causal relationship between symptoms and physiological factors will be discovered by causal discovery

R Ganz (Siebenrock et al., 2010) proposed a surgical plan which can retain the vascular supply of the femoral head, to guarantee the oxygen and nutritional supply to the cells (Kramer et al., 2009). However, excessive vascular supply may be not conducive to recovery. Further research on the pathogenesis or the healing process for femoral head necrosis, especially the causality, but not only the correlation between the symptoms and physiological factors should be deserved. Now causal discovery, which can promote the research of disease mechanism, has been applied in hippocampal function analysis (Mannoor et al., 2013; Sanchez-Romero et al., 2018). Causal discovery can be a useful theory for the research on the pathogenesis or the healing process.

### 3.2 Intelligent technologies will make add the functionality of implants and make the implants manufacture process more efficiently

#### 3.2.1 Implant with intelligent sense will provide real recovery status

**FIGURE 14:**
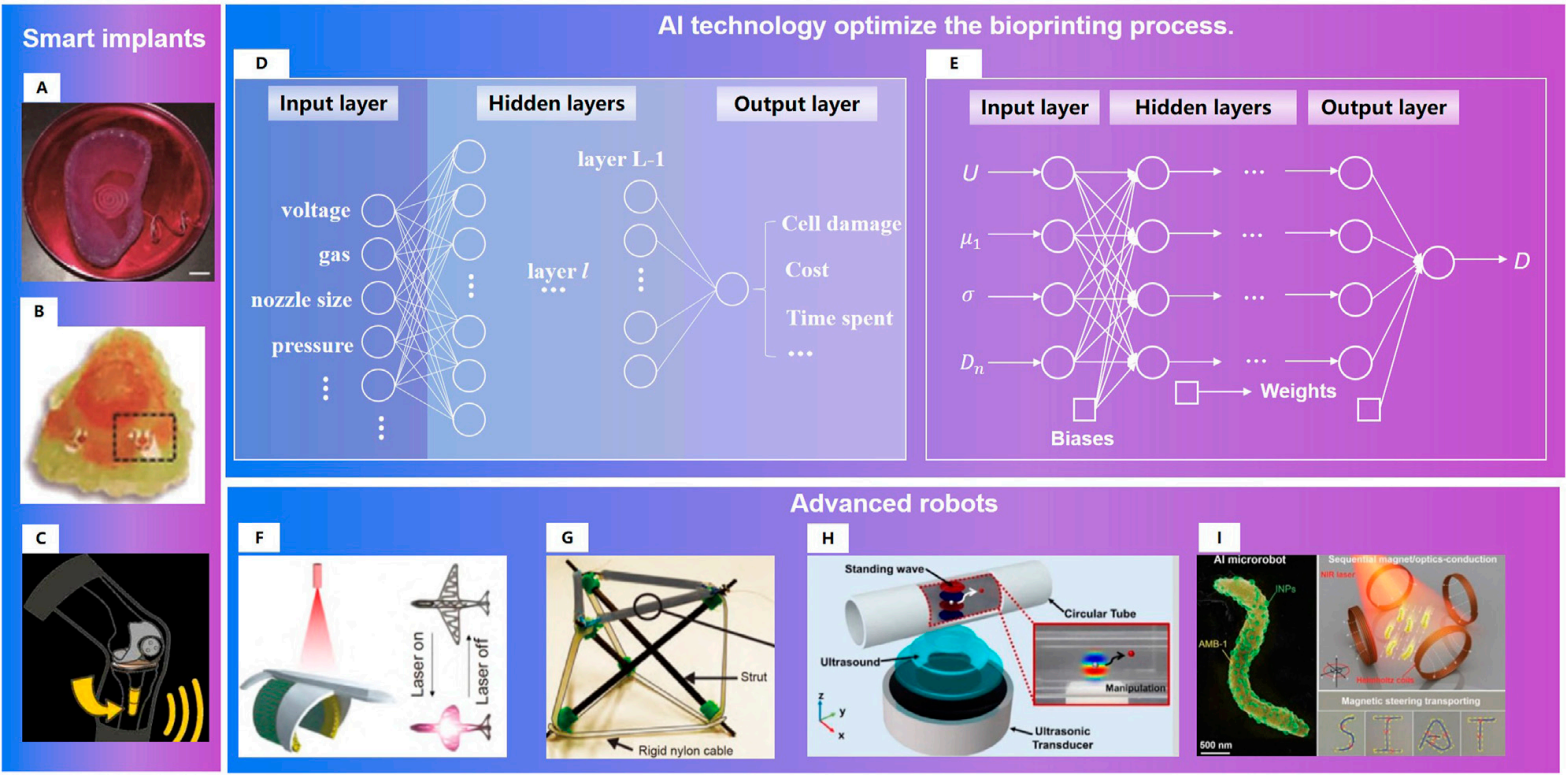
Research directions for satisfied repair of musculoskeletal injuries: 1) Smart implants. **(A)**A bionic ear (Mannoor et al., 2013). **(B)**. An odor-perceptive nose-like hybrid (Jodat et al., 2020). **(C)**. Smart prosthesis (Zimmerbiomet, 2021) 2)AI technology optimize the bioprinting process. **(D)**. Example neural network for process optimization in three-dimensional bioprinting (Ruberu et al., 2021). **(E)**. FCNNs for multi-objective optimization of drop-on-demand (DOD) bioprinting (Shi et al., 2019). 3)Advanced robots. **(F)**. Tissue engineering robot with light activated deformable wing (Xu et al., 2019b). **(G)**. Variable-stiffness tensegrity structures (Zappetti et al., 2020). **(H)**. An ultrasound-controlled actuator for targeted drug delivery (Lee et al., 2020). **(I)**. Acoustic powering and magnetic steering for actuating and navigating microrobots (Aghakhani et al., 2020).

Implants bioprinting combined with intelligent sense is also a research hotspot (Figure 14). In 2013, Mannoor M.S.et al. Mannoor et al. (2013) generated a bionic ear *via* additive manufacturing of biological cells with structural and nanoparticle derived electronic elements. In 2019, Jodat, Y.A. et al. Jodat et al. (2020) designed an odor-perceptive nose-like hybrid, with 3D cartilage-like tissue constructs. It is composed of a mechanically robust cartilage-like construct and a biocompatible biosensing platform. Patients can not recover only by surgery without rehabilitation training as the human body systems has changed after operations. Doctors can obtain the patient’s status through regular follow-up. These data were often temporary and fragmented, and could not truly reflect the patient’s postoperative status. The smart prosthesis persona IQ of Zimmer Biomet has passed FDA certification (Zimmerbiomet, 2021). Persona IQ can not only implant in patients like the traditional prosthesis, but also record the range, speed and other index of gait, for assessing postoperative recovery progress. Implant with intelligent sense will provide real recovery status for changing rehabilitation plans.

#### 3.2.2 Intelligent models 3D/4D printing process will optimize the manufacturing process

The performance of 3d/4d printed matter is not only related to its own physical, mechanical and biological properties, but also affected by the process parameters of the printing process (Naghieh et al., 2020). Study of Zheng W. et al. Zheng et al. (2021) showed the following facts: ① Higher pressures caused instability of extruded biomaterial, and subsequently, poor printability; ②Fell under or above the range of nozzle speeds caused poor strand printability; ③ The more viscous the biomaterial, the more appropriate printability could be achieved. Due to the complex conditions, it is hard to build an accurate mathematical printing model. Data driven AI technology (Shi et al., 2019; Yu and Jiang, 2020; Ruberu et al., 2021) has obvious advantages in the multi-objective optimization of biological printing process (Figure 14).

### 3.3 Advanced materials and variable structures will improve the treatment performance of robotics

As the physiological state of patients are different and changing (Ionov, 2018), such as temperature, pH (Ca2+ concentration), to achieve satisfied repair in long-term therapy, personalized surgical channels should be conducted (Xu et al., 2019b; Zappetti et al., 2020; Wang et al., 2022) or the endogenous repairs should be guided by the micro nano robots (Aghakhani et al., 2020; Lee et al., 2020). Combining the improvement of intraoperative operation skills and the providing signals for postoperative repair, the treatment effect can be improved through robots.

## 4 Conclusion

Satisfied recovery, a key goal of precise orthopaedics, has became possible, as AI technology in orthopaedics has progressed in the following apsects: physical parameters can be predicted precisely through AI technology, bioactive implants made through 3D printing technology can provide signals for endogenous repair, and personalized surgical channels can be established through intelligent robotic technology. However, the multi-layer intelligent technologies (MLIT) has not been used widely. With further study on biomechanical models for multi-physical parameter prediction, stimulus response mechanism of bioactive implants, smart implants, intelligent modeling of 3D/4D printing process and variable structure in long-term therapy (Senatov et al., 2016; Lin et al., 2019; Su et al., 2019), satisfied repair may be achieved by the multi-layer intelligent technologies (MLIT).
